# Remote Sensing Image Dehazing via RGB-Space Physical Constraints

**DOI:** 10.3390/s26134026

**Published:** 2026-06-25

**Authors:** Minxian Shen, Xucong Jiang, Chenyang Shao, Houzheng Zhang, Mingye Ju

**Affiliations:** School of Internet of Things Engineering, Nanjing University of Posts and Telecommunications, Nanjing 210023, China

**Keywords:** RGB-space physical constraints, atmospheric scattering model, atmospheric light estimation, transmission estimation, remote sensing image dehazing

## Abstract

Haze commonly degrades visible-spectrum remote sensing (RS) images by reducing contrast and distorting colors. Existing RS dehazing methods still face two limitations. Prior-driven methods rely on handcrafted assumptions that may become unreliable in complex wide-area scenes without explicit sky regions. Learning-based methods require paired training data, yet real aligned hazy/haze-free RS image pairs are difficult to collect, which limits their real-world generalization. To address these limitations, we propose a method called Remote Sensing Image Dehazing via RGB-Space Physical Constraints (RDPC). The new method revisits the atmospheric scattering model (ASM) from the perspective of RS imaging and builds the restoration process on several physical properties of hazy image formation. For atmospheric light estimation, the RGB-space line-convergence behavior of local regions with similar reflectance and slight depth variations is exploited, allowing atmospheric light to be estimated without explicit sky areas. For transmission estimation, the geometric relation between observed pixels and atmospheric light is used in RGB space, where local perpendicularity provides physically plausible haze-removal guidance and global compensation helps avoid excessive darkening and color degradation. The estimated transmission and albedo guidance are further refined by enforcing ASM consistency and variation sparsity through joint optimization. Experiments on synthetic and real-world RS image dehazing benchmarks demonstrate that RDPC achieves competitive performance against representative prior-based and learning-based methods, including Image Dehazing and Exposure (IDE), Iterative Predictor-Critic (IPC), Curvature-to-Plane Prior (C2P), Adaptive Structure-Texture Awareness (ASTA), Asymmetric U-Net (AU-Net), Efficient Multi-scale Prior Fusion (EMPF), and Lightweight Feature Dehazing (LFD), in terms of peak signal-to-noise ratio (PSNR), structural similarity index measure (SSIM), learned perceptual image patch similarity (LPIPS), Blind/Referenceless Image Spatial Quality Evaluator (BRISQUE), neural image assessment (NIMA), and processing time.

## 1. Introduction

**Figure 1:**
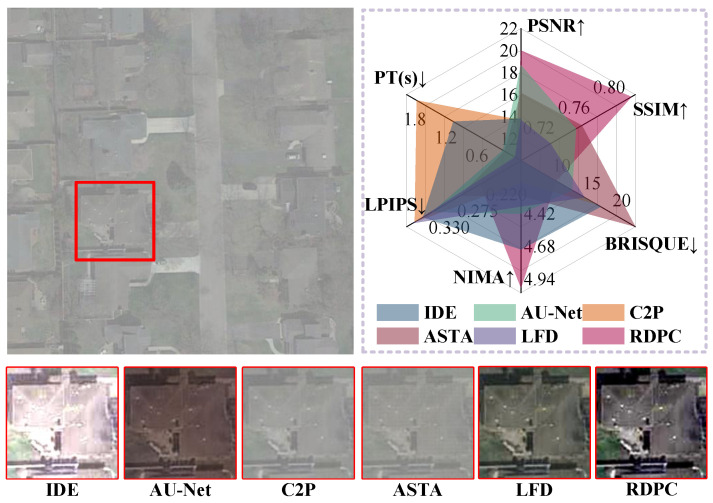
Qualitative and quantitative comparisons between the proposed RDPC and representative dehazing methods, including IDE, AU-Net, C2P, ASTA, and LFD. The red box marks the selected region, and the bottom row shows the zoom-in patches.

Remote sensing (RS) images provide essential observations for many Earth observation tasks, such as environmental monitoring, disaster assessment, and resource exploration [1]. In practical imaging conditions, however, haze introduces atmospheric scattering effects, which reduces contrast, weakens fine details, and causes color distortion [2], as shown in Figure 1. This problem is more difficult in RS imagery because the imaging range is usually large, the land-cover distribution is complex, and the observation distance is long. Once haze contamination appears, the degradation is not limited to visual quality; it may also reduce the reliability of downstream RS interpretation tasks, including land-cover classification [3], change detection [4], object detection [5], and semantic segmentation [6], which depend heavily on accurate structural details, spectral/radiometric consistency, and local contrast. Recent object detection-oriented scene understanding studies further show that high-level perception is highly sensitive to complex scene layouts, object density, weather variations, and domain shifts. For example, RSUD20K [7] provides a road-scene understanding benchmark for autonomous driving and evaluates object detection performance under diverse road and weather conditions, indicating the importance of robust visual inputs for reliable downstream perception. Therefore, reliable and efficient RS image dehazing remains an important problem for both image restoration and practical Earth observation applications. However, existing RS image dehazing methods still struggle under complex wide-area RS conditions, mainly because handcrafted priors may become unreliable in heterogeneous land-cover scenes, while learning-based models trained on synthetic hazy/clear pairs often generalize poorly to real atmospheric degradation.

A natural choice for RS image dehazing is to adapt single-image dehazing methods developed for natural outdoor scenes [7,8,9,10,11], because they require only a single degraded observation and avoid temporal alignment, extra sensors, or special acquisition conditions [12,13]. However, RS images differ from natural images in imaging geometry and scene composition. Natural images are often captured from ground-level viewpoints, where sky regions can help estimate atmospheric light, whereas RS images are acquired from airborne or spaceborne platforms with wider coverage, longer imaging distances, complex land covers, and frequently absent sky regions. These differences make natural-scene assumptions less reliable for RS imagery [14]. In particular, the lack of sky cues complicates atmospheric light estimation, while wide-area observations and heterogeneous terrain make transmission estimation more challenging. As a result, directly applying natural-image dehazing models to RGB visible-spectrum RS imagery may lead to residual haze, over-enhancement, or color distortion.

To better accommodate the characteristics of RS imaging, a number of dehazing methods have been developed for RS imagery [15,16,17,18,19,20]. In general, these methods can be divided into prior-driven methods and deep learning-based methods. The former usually estimate atmospheric light and transmission under the atmospheric scattering model (ASM) with handcrafted priors. They are physically interpretable and do not rely on large-scale training data, but their performance is closely related to whether the adopted priors hold in real scenes. For wide-area RS images, land-cover types, imaging distances, haze densities, and illumination conditions often vary greatly. Under such conditions, a single prior may become unreliable, leading to inaccurate atmospheric light or transmission estimation, residual haze, color distortion, over-enhancement, and loss of fine details [21]. These limitations reveal two key challenges in RS image dehazing: unstable priors and weak real-world generalization due to scarce realistic paired data.

To overcome these challenges, we propose RDPC, a physics-driven framework for single-image RS dehazing. Our central hypothesis is that RS haze degradation is not arbitrary, but is constrained by ASM-consistent RGB-space geometry and sparse variations of imaging variables; therefore, robust RS dehazing can be achieved by exploiting these RS-specific physical properties without relying on a single handcrafted prior or large-scale paired training data. Motivated by this hypothesis, RDPC revisits the atmospheric scattering model (ASM) from the perspective of RS imaging and derives three complementary physical properties related to RGB-space line convergence, geometric haze-removal constraints, and ASM-consistent sparse variations of imaging variables. Based on these properties, RDPC estimates atmospheric light in a local-to-nonlocal manner, estimates transmission through local-to-global modeling, and refines physical variables with variation-aware joint optimization. Without relying on a single handcrafted prior or large-scale paired training data, RDPC provides an interpretable and training-free solution for robust RS image restoration. As shown in Figure 1, RDPC achieves competitive and balanced performance against representative dehazing methods in terms of both visual quality and quantitative metrics, with detailed experimental results and analyses provided in Section 4.

The main contributions of this work are summarized as follows:

- We propose RDPC, a physics-driven framework for single-image RS dehazing. Unlike prior-based methods that rely on a single handcrafted assumption or data-driven methods that require large-scale paired samples, RDPC exploits ASM-derived physical cues from local-to-nonlocal and local-to-global perspectives, providing an interpretable and training-free solution for RS image restoration.
- For atmospheric light estimation, we develop a local-to-nonlocal line convergence module. This module is derived from Physical Property 1: under the ASM, pixels from local regions with similar reflectance and slight depth variations tend to form RGB-space lines passing through the atmospheric light. By aggregating such line-convergence cues from reliable local regions, RDPC estimates atmospheric light without depending on explicit sky-region assumptions.
- For transmission estimation, we introduce a local-to-global mechanism based on Physical Property 2. According to the ASM, dehazing can be viewed as moving hazy pixels toward lower-intensity haze-free radiance under the RGB-space constraint defined by the observed pixels and atmospheric light, while retaining scene information as much as possible. The local perpendicularity constraint provides physically plausible restoration directions, and the global compensation strategy reduces excessive darkening and color degradation, leading to more stable transmission estimation in large-area RS observations.
- We further design a joint optimization module to refine transmission and albedo guidance simultaneously. This module follows Physical Property 3, which requires the estimated imaging variables to be consistent with the ASM while exhibiting sparse variations in spatially coherent regions. With variation-aware regularization, the optimization process suppresses unreliable local fluctuations, improves the consistency of physical variables, and preserves land-cover structures during scene radiance recovery.
- Extensive experiments on synthetic and real-world RS dehazing datasets demonstrate the effectiveness of RDPC. Without network training, RDPC achieves competitive restoration performance against representative prior-based and learning-based methods, and shows good generalization across different haze densities, land-cover types, and imaging conditions.

## 2. Related Work

Existing dehazing methods can be reviewed from two closely related perspectives: general image dehazing and remote sensing (RS) image dehazing. General image dehazing methods are discussed first because they provide the fundamental ASM-based priors and learning paradigms that have influenced many RS dehazing approaches. However, due to the specific imaging geometry and scene characteristics of RS images, these general methods cannot be directly regarded as sufficient solutions for RS dehazing. Therefore, this section first briefly reviews prior-driven and learning-based general image dehazing methods, and then focuses on prior-driven and learning-based RS image dehazing methods to better clarify the motivation of RDPC.

### 2.1. Prior-Driven General Image Dehazing Methods

Prior-driven methods are usually based on the Atmospheric Scattering Model (ASM), which describes hazy image formation as the combination of attenuated scene radiance and atmospheric light:

(1) I(x)=J(x)·t(x)+A·(1−t(x)),

where *x* denotes the pixel coordinate, I is the observed hazy image, J is the haze-free image or scene radiance, A is the global atmospheric light, and *t* is the transmission map related to scene depth. Under this formulation, image dehazing mainly requires accurate estimation of A and *t*. To solve this ill-posed problem, many handcrafted priors have been proposed for general/natural image dehazing [7,22,23,24,25,26,27,28,29,30,31]. According to their assumptions, these methods can be roughly categorized into pixel-wise [22,23], patch-wise [7,24,25], non-local-wise [26,27], and global-wise [29,30] strategies. Although prior-driven methods are interpretable and training-free, their performance heavily depends on the validity of the handcrafted assumptions. When scene content, illumination, haze density, or color distribution deviates from the assumed conditions, inaccurate atmospheric light or transmission estimation may lead to residual haze, over-enhancement, color distortion, or detail loss. Therefore, these general priors provide useful physical insights, but their assumptions may become less reliable when applied to RS images with absent sky regions, complex land covers, and wide-area non-uniform haze.

### 2.2. Deep Learning-Based General Image Dehazing Methods

With the development of deep learning, data-driven methods have achieved significant progress in natural image dehazing. Instead of manually designing priors, these methods learn haze-related representations or direct haze-to-clear mappings from training data. Existing methods can be broadly divided into paired-supervised methods [10,11,32,33,34,35,36] and unpaired/self-supervised methods [37,38,39,40,41]. Paired-supervised methods learn restoration mappings from hazy/clear image pairs, whereas unpaired or self-supervised methods relax the requirement for paired data by introducing cycle consistency, contrastive learning, physical constraints, or distribution alignment. Benefiting from the strong representation capability of deep networks, learning-based methods often produce visually appealing results and often outperform traditional prior-based methods on standard benchmarks. Nevertheless, their generalization ability is largely constrained by the diversity and quality of training data. When test images contain haze distributions, imaging conditions, or scene structures different from those in the training domain, these methods may suffer from unstable restoration, residual haze, or unnatural color shifts. This limitation becomes more evident when general image dehazing models are directly applied to RS imagery, whose imaging geometry, scene scale, and atmospheric degradation characteristics differ substantially from those of ordinary outdoor images. These observations explain why RS-specific dehazing methods are needed: compared with ordinary outdoor images, RS images have different imaging scales, land-cover compositions, and haze distributions, which require more scenario-aware physical modeling or learning strategies.

### 2.3. Prior-Driven Remote Sensing Image Dehazing Methods

RS image dehazing has attracted increasing attention because clear and radiometrically reliable imagery is important for Earth observation. Compared with natural outdoor images, RS images are usually captured from high-altitude or spaceborne platforms and cover large-scale scenes with complex land-cover distributions, long imaging distances, wide depth ranges, and spatially varying atmospheric conditions. Therefore, haze degradation in RS imagery is often highly non-uniform and strongly influenced by acquisition platforms, imaging heights, spatial resolutions, and atmospheric states. A straightforward solution is to extend prior-driven general dehazing methods to RS images. These methods retain the interpretability of the ASM and estimate atmospheric light and transmission according to physical or statistical priors [42,43,44,45,46,47]. Such a paradigm is attractive because high-quality paired RS dehazing datasets are difficult and expensive to collect. However, directly using general priors in RS scenarios is not always reliable. Sky regions may be absent in many RS images, making atmospheric light estimation difficult. Meanwhile, complex land-cover materials, large imaging ranges, and spatially non-uniform haze may violate the local or global assumptions adopted by conventional priors. As a result, prior-driven RS dehazing methods may suffer from inaccurate atmospheric light estimation, unreliable transmission recovery, and unstable restoration in challenging scenes.

### 2.4. Deep Learning-Based Remote Sensing Image Dehazing Methods

To better handle the complex degradation characteristics of RS imagery, many deep learning-based RS dehazing methods have been proposed in recent years [48,49,50,51,52,53,54,55]. More specifically, these methods usually improve restoration performance by designing RS-oriented network architectures, including multiscale feature fusion [48,49], attention mechanisms [50,55], physics-aware modules [51,52], frequency-domain modeling [53,54], diffusion-based restoration [53,54], and recently Mamba-based architectures for satellite RS single-image dehazing [50]. Compared with traditional prior-driven methods, deep models can learn more flexible haze representations from data and have shown promising performance on synthetic and real-world RS dehazing benchmarks. Despite these advances, deep learning-based RS dehazing methods still face several limitations. First, their performance often depends on large-scale and high-quality paired training datasets, while collecting and annotating paired hazy/clear RS images is costly and difficult in real-world scenarios. Second, since paired training data are often generated synthetically, models may suffer from synthetic-to-real domain gaps and generalize poorly to real RS images with complex haze distributions and land-cover variations. Third, although some methods introduce physical models or priors into network design, many deep models still rely mainly on data-driven feature learning and lack explicit physics-based guarantees for robust atmospheric light and transmission estimation.

Beyond image restoration itself, recent object detection and scene understanding studies have also emphasized the importance of robust visual perception under complex environments. For example, RSUD20K [7] provides a road-scene understanding benchmark for autonomous driving, where object detection performance is evaluated under diverse road layouts, traffic-object distributions, and challenging weather conditions. Such studies indicate that high-level perception models are sensitive to scene complexity, image quality, and domain variations. Therefore, although RDPC does not aim to design a new object detector, downstream object detection is included as an auxiliary evaluation to examine whether the restored images can provide more reliable structural and visual cues for high-level RS interpretation.

### 2.5. Discussion

In summary, general image dehazing methods provide useful physical models and learning paradigms, but their assumptions may not fit the imaging characteristics of RS imagery. Existing RS-oriented methods improve scene adaptability, yet prior-driven methods are often limited by unstable handcrafted assumptions, while deep learning-based methods depend on paired data and may suffer from domain gaps. For ASM-based dehazing, atmospheric light and transmission estimation methods [7,26,56,57] remain unreliable when sky regions are absent, land covers are complex, and haze distribution varies across wide-area observations. Motivated by these limitations, we propose RDPC, a physics-driven and interpretable framework for single RS image dehazing. RDPC revisits the ASM from the perspective of RS imaging and exploits complementary physical properties of hazy image formation, rather than relying on a single empirical prior or large-scale retraining. These properties are further embedded into atmospheric light estimation, transmission estimation, and scene radiance recovery, enabling a training-free solution for robust RS image restoration.

## 3. Proposed Methodology

**Figure 2:**
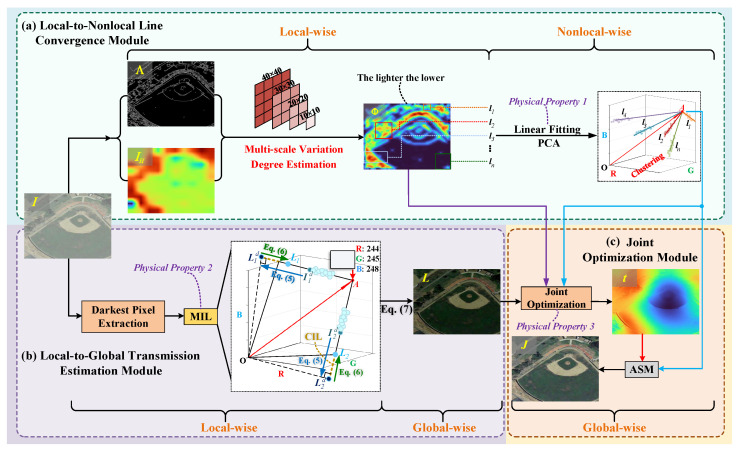
Flow chart of the proposed RDPC. Physical Property 1 enables local-to-nonlocal atmospheric light estimation, Physical Property 2 supports local-to-global transmission estimation, and Physical Property 3 introduces variation-aware joint optimization for refining transmission and albedo guidance.

In this section, we introduce RDPC, a physics-driven framework for single remote sensing (RS) image dehazing. As illustrated in Figure 2, the framework is organized around three ASM-derived physical properties, each corresponding to a specific component of the dehazing process. First, the local-to-nonlocal line convergence module estimates atmospheric light by exploiting RGB-space line-convergence cues from reliable local regions, avoiding the need for explicit sky regions. Second, the local-to-global transmission estimation module combines local perpendicularity with global compensation to guide haze removal while reducing information loss. Third, the joint optimization module refines transmission and albedo guidance under ASM consistency and variation-aware regularization. In this way, RDPC integrates atmospheric light estimation, transmission estimation, and scene radiance recovery into an interpretable and training-free framework for robust RS image dehazing.

### 3.1. Local-to-Nonlocal Line Convergence Module

#### 3.1.1. Motivation of Physical Property 1

**Figure 3:**
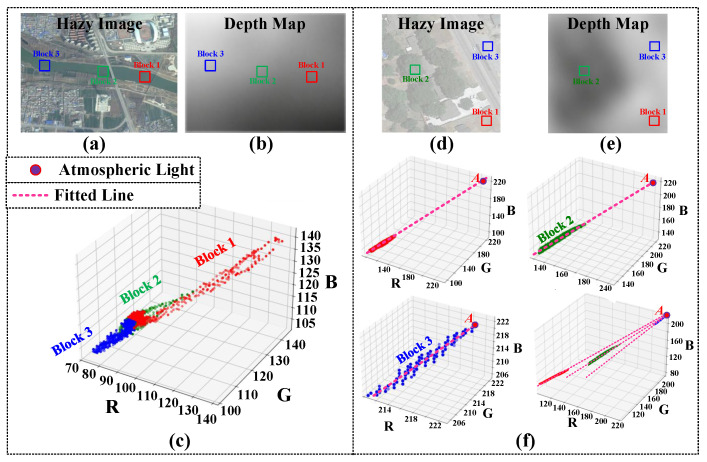
Illustration of Physical Property 1. (**a**,**d**) Hazy images with selected local homogeneous blocks marked by colored boxes. (**b**,**e**) Depth maps corresponding to (**a**,**d**), respectively. (**c**,**f**) RGB-space color distributions of pixels from the corresponding blocks in (**a**,**d**), respectively, where lines with different colors correspond to blocks marked by boxes of the same colors. The projections of the three blocks from (**d**) onto the same plane are shown in (**f**).

In real-world hazy RS images, local blocks often contain pixels with similar reflectance but slight depth variations are frequently observed, and we refer to this observation as Physical Property 1. This is common in RS scenes, where homogeneous land-cover regions often cover a certain spatial extent and thus contain small imaging-distance differences. Figure 3 illustrates the motivation of Physical Property 1 in a more intuitive manner. In Figure 3a,d, several local homogeneous blocks are selected from hazy RS images. Their corresponding depth maps in Figure 3b,e show that these blocks contain slight depth variations, although their material reflectance is relatively consistent. According to the atmospheric scattering model in Equation (1), the observed intensities of such local blocks are constrained by the atmospheric light A and can be expressed as

(2) $$I_{i}\left(x\right)=\left(J_{i}\left(x\right)-A\right)\cdot t_{i}\left(x\right)+A,\;x\in \Omega_{i},$$

where $\Omega_{i}$ denotes the *i*-th non-overlapping block satisfying Physical Property 1. Since pixels within $\Omega_{i}$ have similar material reflectance, their scene radiance $J_{i}\left(x\right)$ can be approximately treated as constant. Thus, the variation of $I_{i}\left(x\right)$ is mainly determined by the scalar transmission $t_{i}\left(x\right)$, which changes with scene depth. As a result, these pixels tend to form a line in RGB space. This behavior is visualized in Figure 3c,f, where the fitted lines of different blocks tend to pass through or converge near the atmospheric light A. Based on Physical Property 1, RDPC estimates atmospheric light by selecting reliable local homogeneous blocks and aggregating their non-local line-convergence cues, thereby avoiding the dependence on explicit sky regions or dense haze regions in RS images.

Here we remark that this RGB-space linear behavior is related to the Color Lines model [58] in natural image dehazing. Different from directly adopting this prior, RDPC exploits it from an RS-specific ASM perspective by selecting local homogeneous RS blocks with slight depth variations and aggregating their non-local line-convergence cues for atmospheric light estimation.

#### 3.1.2. Reliable Block Selection for Atmospheric Light Estimation

To accurately estimate the atmospheric light A based on Physical Property 1, the first step is to identify local blocks that conform to this property. To this end, RDPC designs a multi-scale block searching strategy to locate reliable homogeneous regions in hazy RS images. Specifically, the input hazy image I is first partitioned into non-overlapping blocks with sizes of 10×10, 20×20, 30×30, and 40×40, so that homogeneous regions can be captured at different spatial scales. For convenience, the set of pixel indices belonging to the *i*-th block at the n×n scale is denoted as $B_{i}^{n\times n}$.

A reliable line-convergence block is expected to exhibit sparse texture responses and high color consistency. Therefore, Canny edge detection is applied to I to obtain a binary edge map Λ, which measures the texture density within each block, while the variance of the normalized chromaticity distribution $I_{H}$ is used to evaluate color consistency. Based on these two criteria, the variation degree assigned to pixels in $B_{i}^{n\times n}$ is defined as

(3) $$\varphi^{n}\left(x\right)=\theta \cdot \mathrm{Var}\left(I_{H}\left(B_{i}^{n\times n}\right)\right)+\sum_{j\in B_{i}^{n\times n}}\frac{\Lambda (j)}{n\times n},\;x\in B_{i}^{n\times n},$$

where Var(·) denotes the variance operator. The parameter θ balances the chromaticity-variance term and the texture-density term, and is empirically set to 1.5 in all experiments. The final variation degree map is then computed as

(4) $$\Phi \left(x\right)=\Gamma \left(\sum_{n}n\cdot \varphi^{n}\left(x\right)\right),\;n\in \left\{10,20,30,40\right\},$$

where Γ(·) denotes the standard min-max normalization operation [59], which maps the aggregated multi-scale variation response into a comparable range for reliable block selection. A smaller value of Φ(x) indicates lower local variation, suggesting that the corresponding region is more texture-sparse and color-consistent, and is therefore more suitable as a line-convergence candidate. Finally, RDPC searches for the 10 blocks with the smallest variation degrees using sliding windows across different spatial scales. To avoid selecting redundant blocks with similar RGB-space line directions, the angles between their fitted lines $l_{i}$ are constrained to be larger than 15°. After the candidate blocks are selected, principal component analysis (PCA) is employed to fit an RGB-space line for each block. The intersections of these fitted lines are then computed, and the clustering center of the intersection points is taken as the atmospheric light A. This strategy also helps reduce the influence of unreliable blocks caused by complex land covers. Blocks containing strong textures, mixed materials, or obvious structural boundaries usually have larger variation degrees and are therefore less likely to be selected. Moreover, the angular constraint between fitted RGB-space lines and the non-local aggregation of multiple line intersections further reduce the effect of redundant or unstable candidates on atmospheric light estimation. In this way, the proposed module follows a local-to-nonlocal strategy: reliable local homogeneous blocks are first selected, and their non-local line-convergence cues are then aggregated for robust atmospheric light estimation, without requiring explicit sky regions or dense haze regions as atmospheric-light candidates. For clarity, the overall atmospheric light estimation process is illustrated in Figure 2a. Specifically, in Figure 2a, the edge map and chromaticity consistency are used to compute the multi-scale variation degree map in Equations (3) and (4), where brighter regions indicate lower local variation and are more suitable for reliable block selection. The selected local blocks are then fitted by PCA in RGB space, and their non-local line-convergence cues are aggregated to estimate the atmospheric light A.

### 3.2. Local-to-Global Transmission Estimation Module

#### 3.2.1. Motivation of *Physical Property 2*

The essence of image dehazing is to recover the latent haze-free image J by suppressing haze-induced brightness while preserving scene information. Under the ASM, the observed hazy pixel I(x), the latent scene radiance J(x), and the atmospheric light A are constrained along the same RGB-space line [7]. Therefore, dehazing should move I(x) away from A toward a lower-intensity position along this physical line, rather than modifying its RGB values arbitrarily. In RGB space, a shorter distance to the origin generally indicates lower pixel intensity. Thus, the perpendicular foot from the origin to the line determined by A and I(x) can be regarded as the lowest-intensity reference on this line. However, computing such a reference for every pixel in a local block would introduce unnecessary computational cost and may be sensitive to local noise. Therefore, local darkest pixels are used as representative cues for the lower-intensity direction of latent radiance. The perpendicular foot from the origin to the line determined by A and the local darkest pixel then serves as a physically constrained haze-removal reference. This perpendicular guidance suppresses haze-induced brightness while preserving the ASM-induced geometric relationship, thereby alleviating information loss and color distortion. We refer to this perpendicular-guidance principle as Physical Property 2.

#### 3.2.2. Local Perpendicularity and Global Compensation for Transmission Estimation

Following Physical Property 2, RDPC estimates the transmission map through a local-to-global strategy, whose overall procedure is illustrated in Figure 2b. The local stage constructs perpendicular guidance from local darkest pixels, while the global stage compensates this guidance to avoid excessive darkening and color information loss. Specifically, the darkest point $I_{i}^{d}$ is first extracted from each local patch, which is related to the local darkest-pixel observation used in the Dark Channel Prior (DCP) [7]. Different from DCP, RDPC does not directly use the dark-channel assumption for transmission estimation. Instead, the local darkest pixel $I_{i}^{d}$ is used as a representative low-intensity cue to construct the RGB-space line determined by A and $I_{i}^{d}$. Under the ASM, the corresponding haze-free radiance should lie on this line. Therefore, RDPC searches for a lower-intensity guidance point along this ASM-induced line, rather than modifying RGB values in an arbitrary direction. The perpendicular foot from the origin to this line is then taken as the local haze-removal guidance $L_{i}^{d}$, which is termed minimum-information-loss (MIL) guidance under the proposed geometric constraint. The detailed derivation is provided in the Supplementary Material. Accordingly, $L_{i}^{d}$ is computed as

(5) $$L_{i}^{d}=A-\frac{A\cdot (I_{i}^{d}-A)}{∥I_{i}^{d}{-A∥}_{2}^{2}}\left(I_{i}^{d}-A\right),$$

where · denotes the dot product. However, directly using $L_{i}^{d}$ as albedo guidance may be unstable, since the perpendicular foot can fall outside the first octant of RGB space. In such cases, the guidance may become excessively dark or even physically invalid, leading to over-enhancement and color information loss. The detailed derivation of Equation (5) is provided in the Supplementary Material.

To address this problem, RDPC introduces a global compensation strategy that adaptively moves $L^{d}$ toward the atmospheric light A, as indicated by the green arrows in Figure 2b. This compensation is designed to reduce the color loss caused by overly dark or physically invalid perpendicular guidance, and is therefore referred to as color loss compensation (CIL). Specifically, the compensation coefficient *R* is determined by shifting $L^{d}$ toward the atmospheric light *A* until all compensated RGB values become non-negative, with the minimum channel value reaching the zero boundary when compensation is required:

(6) $$R^{*}=\min\left\{R\in \left[0,1\right]\;|\;\min_{i\in \omega ,\;c\in \{r,g,b\}}\left(L_{i,c}^{d}+R\left(A_{c}-L_{i,c}^{d}\right)\right)\geq 0\right\},$$

where *i* denotes the pixel/block index, *c* denotes the color channel index, ω denotes the index set of the whole image, and $R^{*}$ is the compensation coefficient. With this compensation, the albedo guidance *L* is obtained by

(7) $$L=R^{*}\cdot A+\left(1-R^{*}\right)\cdot L^{d}.$$

Finally, the rough transmission is estimated according to the relative distance between the darkest hazy pixel and the compensated albedo guidance in RGB space:

(8) $$t_{i}\left(x\right)=\frac{∥I_{i}^{d}{-A∥}_{2}}{∥A-L_{i}{∥}_{2}},\;x\in \Omega_{i}.$$

In this manner, Physical Property 2 is implemented through a local-to-global transmission estimation strategy. The local perpendicularity constraint provides physically plausible lower-intensity guidance for haze removal, and the global compensation term prevents excessive correction. Consequently, RDPC obtains a more stable transmission map while preserving color and structural information. In Figure 2b, the blue arrows denote the MIL-guided movement from local darkest pixels to their perpendicular feet, corresponding to Equation (5), while the green arrows denote the CIL-based global compensation toward A, corresponding to Equations (6) and (7). The compensated guidance is then used to estimate transmission according to Equation (8).

Here we note that the use of local darkest pixels is related to the Dark Channel Prior (DCP) [7]. However, RDPC does not directly use the dark-channel assumption for transmission estimation. Instead, the darkest pixel is used only as a representative low-intensity cue within a local patch, from which RDPC constructs RGB-space perpendicular guidance and global compensation for transmission estimation.

### 3.3. Joint Optimization Module

Although the local-to-global transmission estimation module provides a reasonable initial estimate, local inconsistencies may still persist in the transmission map and albedo guidance, especially near depth discontinuities, object boundaries, and regions with complex color variations. In addition, the estimated physical variables should be consistent with the ASM and should not fluctuate unnecessarily in spatially coherent regions. We define this requirement of ASM consistency and variation sparsity as Physical Property 3. Based on this property, RDPC refines the transmission map and albedo guidance through the following joint optimization:

(9) $$\begin{array}{c} \underset{\left\{t,L\right\}}{\mathrm{minimize}}\frac{\lambda }{2}{∥L\cdot t+\left(1-t\right)\cdot A-I^{d}∥}_{2}^{2}\\ \;\;\;+\frac{\alpha }{2}\Upsilon \left(t\cdot \left(1-\Phi \right)\right)+\frac{\beta }{2}\Psi \left(L\cdot \left(1-\Phi \right)\right), \end{array}$$

where the first term enforces consistency with the atmospheric scattering model, Y(·) regularizes the transmission map to improve spatial coherence, and Ψ(·) regularizes the albedo guidance to suppress unreliable fluctuations. The coefficients λ, α, and β are set to 0.3, 0.7, and 0.5, respectively.

In Equation (9), the variation degree map Φ controls the strength of regularization at different image locations. For regions with low variation degree, the regularization terms encourage smoother transmission and albedo guidance. For regions with high variation degree, such as edges or structural boundaries, the data fidelity term plays a stronger role to avoid over-smoothing. In this way, the optimization process improves the consistency of the estimated physical variables while preserving important land-cover structures. Equation (9) is a total variation optimization problem and is approximately solved by variable splitting and alternating optimization [60,61]. After obtaining the refined transmission *t*, the haze-free image J is recovered as

(10) $$J=\frac{I-A}{\min(\max(t,0.01),1)}+A.$$

With this module, the physical variables obtained from atmospheric light estimation and transmission estimation are further refined. The joint optimization process is illustrated in Figure 2c. As shown in Figure 2c, the estimated transmission and albedo guidance are jointly refined by Equation (9), where the ASM consistency term constrains the physical reconstruction process and the variation-aware regularization terms suppress unreliable fluctuations in spatially coherent regions before recovering the haze-free image. The complete RDPC pipeline, including local-to-nonlocal atmospheric light estimation, local-to-global transmission estimation, and variation-aware joint optimization, is shown in Figure 2.

## 4. Experiments

In this section, we evaluate the proposed RDPC from multiple perspectives. We first describe the experimental settings, including parameter settings, datasets, compared methods, implementation details, and evaluation metrics. Then, the accuracy of atmospheric light estimation and the effectiveness of key components are examined through dedicated evaluation and ablation studies. Finally, RDPC is compared with state-of-the-art methods on real-world and synthetic RS hazy images, followed by evaluation of downstream object detection and analysis of limitations.

### 4.1. Parameter Settings

The parameters of RDPC are fixed across all experiments. This setting is adopted to ensure fair comparison and avoid dataset-specific parameter tuning. In the local-to-nonlocal line convergence module, multi-scale blocks of 10×10, 20×20, 30×30, and 40×40 are used to capture homogeneous regions at different spatial scales. These block sizes are chosen because RS images usually contain both small local structures and large land-cover regions, and a multi-scale search is therefore more suitable than using a single block size. The 10 blocks with the smallest variation degrees are selected as candidates, which provides sufficient line-convergence evidence while avoiding excessive unreliable candidates and computational cost. The angles between their fitted RGB-space lines are constrained to be larger than 15° to avoid unstable intersections. This threshold is used to reduce redundancy among fitted lines and maintain the diversity of the selected candidates in RGB space. The parameter θ is set to 1.5 to balance chromaticity variance and texture density. In the joint optimization module, λ, α, and β are fixed to 0.3, 0.7, and 0.5, corresponding to the data fidelity, transmission regularization, and albedo-guidance regularization terms, respectively. These weights are empirically selected to balance haze removal, radiance fidelity, and structural regularization. In practice, overly large regularization weights may over-smooth image details, whereas overly small weights may lead to residual haze or unstable transmission estimation. Once determined, all these parameters are kept unchanged for different datasets and experimental settings.

### 4.2. Experimental Settings

To evaluate RDPC comprehensively, we conduct experiments on atmospheric light estimation, component ablation, dehazing quality assessment, and downstream object detection. These experiments examine the method from different aspects, including restoration fidelity, structural preservation, perceptual quality, robustness, and practical applicability.

**Datasets.** Four RS dehazing datasets are used in the experiments, including RSHaze [62], StateHaze1K [12], LHID [63], RRSD300 [64], and DHID [49]. Among them, RSHaze, StateHaze1K, and LHID are synthetic or virtually degraded datasets with paired hazy/clear images, which enable objective quantitative evaluation using full-reference metrics. Specifically, RSHaze is adopted to evaluate the behavior of different methods under more realistic synthetic haze degradation, while StateHaze1K and LHID provide paired samples for comprehensive quantitative comparison. In contrast, RRSD300 consists of real-world hazy RS images, where haze-free ground truths are generally unavailable. Therefore, it is mainly used for qualitative comparison to further examine the robustness and practical applicability of RDPC in real RS scenes with different haze levels.

**Compared Methods.** RDPC is compared with several representative dehazing methods, including IDE [31], ASTA [18], AU-Net [17], C2P [11], EMPF [16], IPC [33], and LFD [15]. These methods cover prior/model-driven approaches, deep learning-based restoration methods, and RS-oriented dehazing frameworks, providing a diverse set of baselines for comparison.

**Implementation Details and Metrics.** All experiments are performed on a workstation with an Intel(R) Core(TM) i9-12900KF CPU (Intel Corporation, Santa Clara, CA, USA) and an NVIDIA GeForce RTX 3080Ti GPU (NVIDIA Corporation, Santa Clara, CA, USA). The proposed RDPC was implemented in Python 3.12.3, and the downstream object detection experiments were conducted using the Ultralytics YOLO package, version 8.4.30, including YOLOv8 and YOLOv8-OBB. For fairness, competing methods are tested on the same hardware platform whenever possible, and their parameters follow the original papers or public implementations. The parameters of RDPC are fixed across all experiments unless otherwise stated. Peak signal-to-noise ratio (PSNR) [65] and structural similarity (SSIM) [65] are used to evaluate reconstruction fidelity and structural consistency, while Learned Perceptual Image Patch Similarity (LPIPS) [66] is adopted to measure perceptual similarity. In addition, two no-reference image quality assessment metrics, namely Blind/Referenceless Image Spatial Quality Evaluator (BRISQUE) [67] and Neural Image Assessment (NIMA) [68], are introduced to evaluate the perceptual quality and naturalness of restored images without relying on ground-truth references. For PSNR, SSIM, and NIMA, higher values indicate better performance, whereas lower LPIPS and BRISQUE values indicate better perceptual quality. The average processing time (PT) is reported to evaluate computational efficiency. Together, these metrics provide a comprehensive assessment of restoration accuracy, structural preservation, perceptual quality, naturalness, and efficiency.

### 4.3. Atmospheric Light Estimation Accuracy

**Figure 4:**
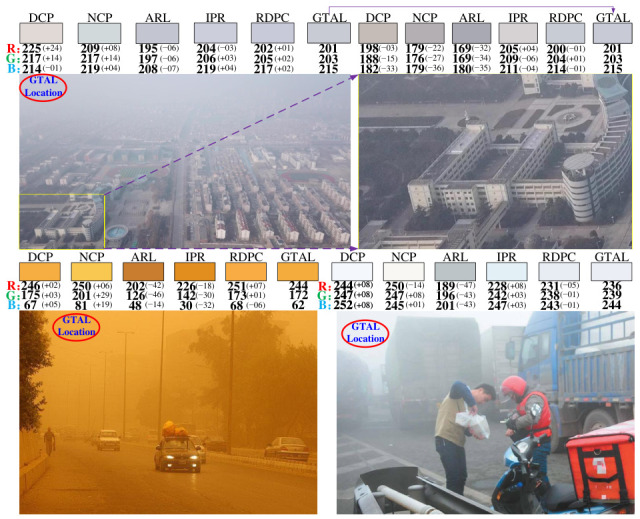
Comparison of atmospheric light estimated by DCP, NCP, ARL, IPR, and RDPC, along with the manually extracted ground-truth atmospheric light (GTAL). The color patches and RGB values indicate the atmospheric light estimated by different methods. In the top RS example, the right image is a zoomed-in view of the yellow boxed region in the left image. The red ellipse marks the manually selected GTAL location. The bottom examples are general outdoor hazy images, where the GTAL locations are also manually annotated.

RDPC adopts a local-to-nonlocal line convergence strategy for atmospheric light estimation. To validate its effectiveness, we compare RDPC with DCP [7], NCP [26], ARL [69], and IPR [28], and use the manually extracted ground-truth atmospheric light (GTAL) as reference, as shown in Figure 4. The red ellipses indicate the manually selected GTAL locations, and the top-right panel provides a zoomed-in view of the yellow boxed region in the top-left RS image.

As shown in Figure 4, the compared methods show significant deviations in estimation in different scenes. In the RS example, where explicit sky regions are absent and the scene contains complex land-cover structures, DCP, NCP, ARL, and IPR produce atmospheric light values that deviate from GTAL to varying degrees. This is because atmospheric light estimation in RS images is easily affected by high-reflectance land-cover regions, such as buildings, roads, bare soil, and clouds. These regions may be mistakenly regarded as atmospheric-light candidates by methods that rely on bright pixels, dark-channel statistics, or dense-haze assumptions. Similar limitations can also be observed in prior-based RS dehazing methods such as IDRLP [56] and IHDCP [57], whose atmospheric light estimation may become less stable when complex surface materials or non-sky bright regions are present. Similar instability can also be observed in the general outdoor hazy images, especially under dense haze or strong color bias. In contrast, RDPC obtains atmospheric-light estimates that are closer to GTAL in most RGB channels, as indicated by the smaller channel-wise deviations highlighted in red in Figure 4. The reason is that RDPC does not directly select atmospheric light from bright pixels or predefined haze-dense regions. Instead, it first identifies locally homogeneous and color-consistent blocks at multiple spatial scales, and then aggregates their non-local RGB-space line-convergence cues. Under the atmospheric scattering model, reliable homogeneous regions tend to generate RGB-space lines that converge toward the atmospheric light, making the clustering center of their intersections a more stable estimate. In addition, the multi-scale block search and line-angle constraint help suppress redundant or unreliable land-cover regions. This verifies the effectiveness of Physical Property 1: by selecting reliable local homogeneous regions and aggregating their non-local RGB-space line-convergence cues, RDPC can estimate atmospheric light without relying on explicit sky regions or dense haze regions as atmospheric-light candidates.

### 4.4. Ablation Study

Since the atmospheric light estimation module has been evaluated in Section 4.3, this ablation study focuses on the refinement stage in the joint optimization module. Specifically, we evaluate two key regularization terms that affect the stability of the estimated physical variables: the albedo guidance (AG) term and the refined transmission (RT) term. The AG term regularizes the albedo guidance L to suppress unstable local variations, whereas the RT term regularizes the transmission map *t* to improve spatial coherence. To examine their individual and joint effects, four variants are compared on the LHID dataset under the same experimental settings: w/o AG, w/o RT, w/o AG&RT, and the full RDPC. Specifically, w/o AG removes the albedo-guidance regularization term, w/o RT removes the transmission regularization term, and w/o AG&RT removes both terms.

**Figure 5:**
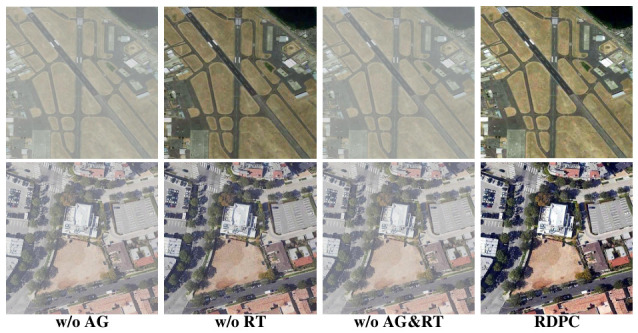
Ablation study on the proposed RDPC.

Table 1 reports the quantitative results on LHID, and Figure 5 presents the corresponding visual comparisons. As shown in Table 1, the complete RDPC model achieves superior overall performance, with the highest PSNR and SSIM and the lowest LPIPS. When the AG term is removed, PSNR decreases from 20.31 to 18.12 and LPIPS increases from 0.1996 to 0.2314, indicating that albedo-guidance regularization is important for maintaining scene appearance and perceptual quality. When the RT term is removed, the performance also drops noticeably, with PSNR decreasing to 17.31 and SSIM decreasing to 0.7576, which suggests that transmission regularization contributes to stable haze suppression and structural consistency. Removing both AG and RT leads to the worst performance, with PSNR, SSIM, and LPIPS degrading to 15.57, 0.6776, and 0.2799, respectively. This confirms that the two terms play complementary roles in the joint optimization module. The visual results in Figure 5 are consistent with the quantitative results. Without both AG and RT, the restored images still contain obvious residual haze and exhibit weak contrast. Removing either AG or RT also degrades visibility and detail recovery to different degrees. In contrast, the full RDPC produces clearer structures, more natural illumination, and better contrast, especially around building contours, facade details, and street-level objects. These comparisons demonstrate that the AG term mainly improves albedo-guided restoration and appearance preservation, while the RT term enhances the stability of transmission refinement and helps suppress residual haze. With both terms included, RDPC achieves a better balance among haze removal, structure preservation, and perceptual quality on LHID.

### 4.5. Comparison with State-of-the-Art Methods

#### 4.5.1. Comparison on Real-World Hazy Images

**Figure 6:**
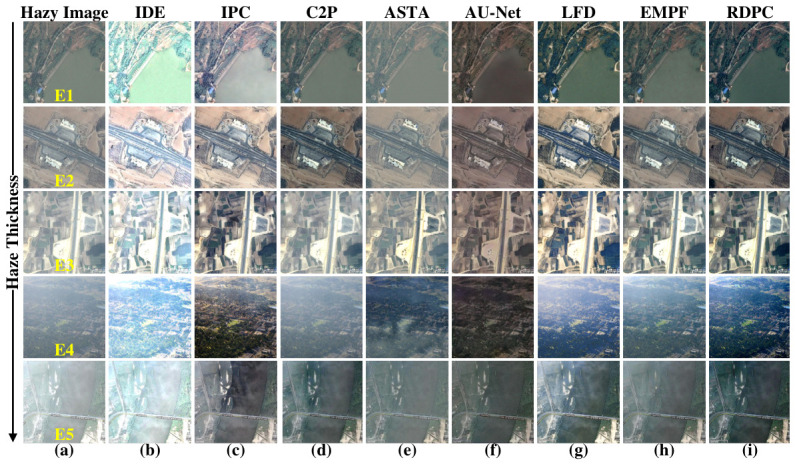
Qualitative comparison between RDPC and other state-of-the-art methods on real-world hazy RS images. (**a**) Hazy Image. (**b**) IDE. (**c**) IPC. (**d**) C2P. (**e**) ASTA. (**f**) AU-Net. (**g**) LFD. (**h**) EMPF. (**i**) RDPC.

To evaluate practical dehazing performance, RDPC is compared with seven representative methods on five representative real-world RS images from RRSD300 [64], as shown in Figure 6. These images contain different haze characteristics and thus provide a more realistic testbed for examining the robustness of RDPC in practical RS dehazing scenarios. Overall, the competing methods show different weaknesses. IDE removes part of the haze, but residual haze remains in E1 and E3, and color over-enhancement appears in E4. IPC recovers some details but introduces noticeable color shifts. C2P is less effective in dense-haze regions, whereas ASTA tends to produce dim results. AU-Net improves visibility to some extent, but its outputs are often dark and brownish. LFD restores clearer structures in some cases, yet the appearance is not always consistent. EMPF gives relatively balanced results among the compared methods, although residual haze and insufficient detail recovery can still be observed in E1 and E3.

RDPC produces clearer and more natural results across these real-world cases. It restores scene contrast and structural details well in E1, preserves road and building appearances in E2, suppresses color distortion while improving visibility in E3, and achieves a good balance between haze removal and color fidelity in E4. In the non-uniform haze scene E5, most competing methods become unstable: some leave obvious residual haze in heavily degraded areas, such as IDE and C2P around the cloud-like haze regions, while others over-enhance lightly hazed regions or introduce local color distortion, such as LFD and AU-Net around the water surface and urban structures. These results suggest that RDPC is effective in haze removal while preserving realistic appearance under complex real-world RS haze degradation.

#### 4.5.2. Comparison on Synthetic Hazy Images

**Figure 7:**
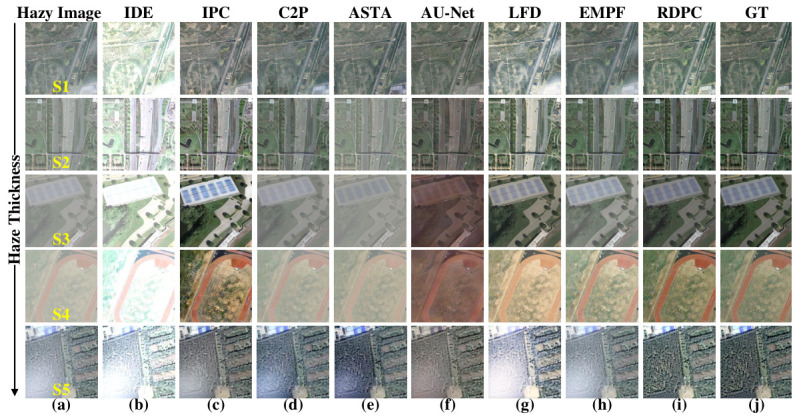
Qualitative comparison between RDPC and other state-of-the-art methods on synthetic hazy RS images. (**a**) Hazy Image. (**b**) IDE. (**c**) IPC. (**d**) C2P. (**e**) ASTA. (**f**) AU-Net. (**g**) LFD. (**h**) EMPF. (**i**) RDPC. (**j**) GT.

StateHaze1K [12] and LHID [63] are further used to compare RDPC with state-of-the-art methods on paired synthetic RS dehazing benchmarks. Figure 7 shows the qualitative results on five challenging examples selected from these datasets. The compared methods exhibit different degradation patterns after restoration. IDE removes part of the haze veil, but residual haze and over-enhanced appearance remain evident, especially in S3 and S4. IPC recovers some local details, yet it often introduces color deviation and unstable tone reproduction. C2P and ASTA tend to produce conservative results, with residual haze and low contrast still visible in several scenes. AU-Net improves visibility to some extent, but its outputs are prone to dark and brownish tones. LFD restores clearer structures in some cases, while over-enhanced brightness and appearance inconsistency can still be observed. EMPF gives relatively balanced results among the competing methods, but residual haze and insufficient structural recovery remain in S2 and S3.

Compared with these methods, RDPC restores clearer scene structures and more natural appearances by exploiting physical cues from local-to-nonlocal and local-to-global perspectives. It better preserves global scene contrast in S1, recovers roofs and surrounding vegetation more naturally in S2, improves visibility while suppressing color distortion in S3, and achieves a better balance between haze removal and structural fidelity in S4. For the non-uniform haze case in S5, most competing methods struggle to handle spatially varying degradation: some fail to remove dense haze regions, such as IDE and C2P in cloud-like degraded areas, while others over-enhance lightly degraded areas and introduce local color shifts, such as LFD and AU-Net around relatively clear land-cover regions. In contrast, RDPC suppresses uneven haze more consistently while preserving local contrast and land-cover structures. Since StateHaze1K and LHID provide paired references, these synthetic comparisons complement the real-world experiments by directly examining whether the restored structures and colors are consistent with the expected haze-free appearance.

#### 4.5.3. Quantitative Comparison

Table 2 reports the average PSNR, SSIM [65], and LPIPS [66] results on RSHaze [62], StateHaze1K [12], and LHID [63]. The processing time (PT) is separately measured on a 512×512 input image for efficiency comparison. As shown in the table, RDPC achieves strong and balanced performance across different datasets and metrics. On RSHaze, RDPC obtains the best PSNR, SSIM, and LPIPS, showing its robustness under more realistic RS haze degradation. On StateHaze1K, ASTA obtains higher PSNR and SSIM values, indicating its advantage in pixel-level fidelity. In contrast, RDPC achieves a better LPIPS score, suggesting improved perceptual similarity and structure-aware visual quality. This reveals a trade-off between fidelity-oriented and perception-oriented evaluation metrics. Since remote sensing image dehazing is not only expected to reconstruct pixel values but also to recover clear structures for visual interpretation and downstream analysis, LPIPS provides a complementary perspective to PSNR and SSIM. Therefore, RDPC is not claimed to be uniformly superior to ASTA on all metrics; instead, it shows advantages in perceptual quality and object-structure preservation, as further supported by the downstream detection results. On LHID, RDPC achieves the best PSNR and the second-best SSIM and LPIPS, demonstrating its advantage in structure preservation and overall restoration quality. In terms of efficiency, RDPC obtains the lowest PT on 512 × 512 inputs, suggesting that the proposed physics-driven method can achieve effective dehazing with low computational cost.

Table 3 further presents the no-reference image quality assessment results on RSHaze [62], StateHaze1K [12], and LHID [63] using BRISQUE and NIMA. Since lower BRISQUE and higher NIMA values indicate better perceptual quality, these metrics provide complementary evaluation beyond full-reference measurements. As shown in Table 3, RDPC achieves the best results on all three datasets for both metrics. Specifically, RDPC obtains the lowest BRISQUE scores of 14.1484, 5.1044, and 13.2968 on RSHaze, StateHaze1K, and LHID, respectively, while also achieving the highest NIMA scores of 5.1373, 5.0615, and 4.8318. These results indicate that RDPC can produce more natural and visually pleasing restoration results without introducing obvious unnatural artifacts.

Overall, the quantitative comparisons in Table 2 and Table 3 demonstrate that RDPC achieves a favorable balance among reconstruction fidelity, structural preservation, perceptual quality, naturalness, and computational efficiency. The improvements indicated by both full-reference and no-reference metrics are also consistent with the qualitative comparisons in Figure 6 and Figure 7, further confirming the effectiveness and generalization ability of the proposed physics-driven dehazing mechanism.

### 4.6. Downstream Object Detection Evaluation

**Figure 8:**
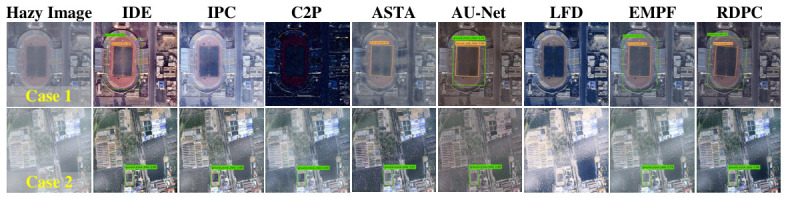
Downstream object detection comparison on a representative hazy remote sensing image. The pretrained YOLOv8 detector is applied to the hazy image and the dehazed results generated by different methods. The confidence scores of correctly detected objects are reported.

**Figure 9:**
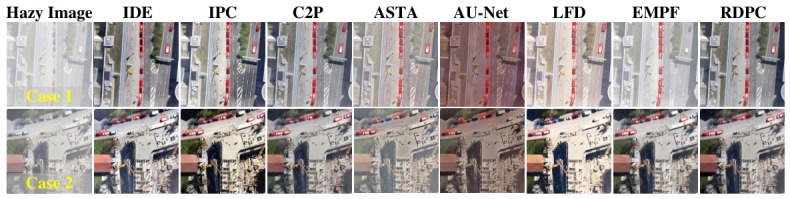
Downstream small-object detection comparison on representative hazy remote sensing image regions. The pretrained YOLOv8-OBB detector is applied to the hazy image and the dehazed results generated by different methods, including IDE, IPC, C2P, ASTA, AU-Net, LFD, EMPF, and RDPC.

To evaluate the practical usefulness of RDPC for high-level RS interpretation, we conduct downstream object detection experiments on two datasets using fixed pretrained detectors. Specifically, YOLOv8 is used for the representative object detection comparison on StateHaze1K in Figure 8 and Table 4, where soccer-ball fields (SBF) and ground-track fields (GTF) are selected as representative targets. In Table 4, “–” indicates that the corresponding target is not detected, and the reported values denote the confidence scores of correctly detected objects. For the whole StateHaze1K dataset, “Avg. SBF” and “Avg. GTF” denote the average detection confidence of soccer-ball fields and ground-track fields, respectively. In addition, YOLOv8-OBB is adopted for the small-object detection comparison on DHID [49] in Figure 9 and Table 5, since oriented bounding boxes are more suitable for small RS objects with arbitrary orientations. In Table 5, “Number” denotes the number of detected objects, and “Conf.” denotes the average confidence of the detected objects in each representative case. For the whole DHID dataset, “Total Number” denotes the total number of detected objects over all test images, whereas “Avg. Conf.” denotes the average confidence of all detected objects. All detectors are directly applied to the hazy and restored images without fine-tuning, under identical inference settings. This setting mainly evaluates the compatibility of the restored images with off-the-shelf detectors and demonstrates the potential of RDPC as a preprocessing module for existing RS interpretation models. However, it does not fully characterize how the dehazing process interacts with detector feature extraction when the detector is explicitly trained or fine-tuned on the restored-image domain, which remains a limitation of the current evaluation.

As shown in Figure 8 and Table 4, haze degradation weakens object boundaries and reduces detection reliability, while different dehazing methods produce different downstream detection responses. On the representative StateHaze1K examples, RDPC achieves the highest confidence for SBF and GTF in Case 1, reaching 0.89 and 0.78, respectively. For the GTF target in Case 2, RDPC obtains a competitive confidence of 0.88, slightly lower than ASTA with 0.91. However, ASTA fails to detect the GTF target in Case 1, suggesting less stable detection performance across different scenes. On the whole StateHaze1K dataset, RDPC achieves the best average confidence for both SBF and GTF, reaching 0.7469 and 0.7285, respectively, outperforming the second-best IDE results of 0.7317 and 0.7146. These results indicate that RDPC can better recover object-related structures and improve the confidence of downstream YOLOv8 detection.

The small-object detection results on DHID are further reported in Figure 9 and Table 5. Compared with common horizontal bounding boxes, YOLOv8-OBB provides oriented boxes that are more suitable for small vehicles and other arbitrarily oriented RS objects. In the two representative DHID cases, RDPC achieves the highest average confidence scores of 0.8062 and 0.7281, respectively. On the whole DHID dataset, RDPC detects the largest number of objects, reaching 320, and also obtains the highest average confidence of 0.7324. In comparison, the second-best IPC detects 305 objects with an average confidence of 0.7216. These results show that RDPC not only improves detection confidence but also helps retain more detectable small-object structures after dehazing. Overall, this evaluation is used as an auxiliary analysis to verify whether dehazing benefits subsequent RS interpretation tasks, rather than as a comprehensive object detection benchmark.

### 4.7. Application Extension

**Figure 10:**
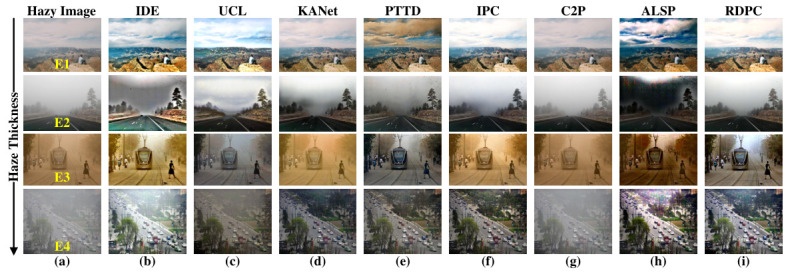
Qualitative comparison between RDPC and other state-of-the-art methods on real-world outdoor hazy images. (**a**) Hazy Image. (**b**) IDE. (**c**) UCL. (**d**) KANet. (**e**) PTTD. (**f**) IPC. (**g**) C2P. (**h**) ALSP. (**i**) RDPC.

Although RDPC is developed for RS image dehazing, the proposed physics-driven mechanism can also be applied to general outdoor hazy images. To examine this extension, RDPC is compared with several representative outdoor dehazing methods, including IDE [31], UCL [39], KANet [34], PTTD [38], IPC [33], C2P [11], and ALSP [70]. As shown in Figure 10, RDPC achieves competitive visual results on real-world outdoor scenes with different haze densities. Compared with the competing methods, RDPC effectively reduces haze while maintaining natural colors and structural details, and avoids obvious over-enhancement in challenging cases. This indicates that the ASM-derived physical cues used in RDPC have good applicability beyond RS imagery, providing a potential training-free solution for broader outdoor dehazing scenarios.

### 4.8. Limitation Analysis

**Figure 11:**
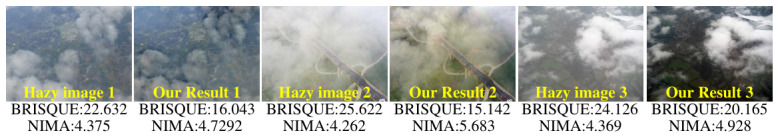
Failure cases of RDPC in cloud–haze coupled scenes. When clouds and haze coexist, RDPC may suffer from residual haze, over-enhancement, or local color distortion due to the visual similarity between clouds and haze and the limited applicability of the atmospheric scattering model.

Although RDPC performs well in most RS dehazing cases, it remains limited in cloud–haze coupled scenes. As shown in Figure 11, residual haze, over-enhancement, or local color distortion may appear around cloud-covered areas. This is because clouds and haze share similar RGB cues, such as high brightness, low saturation, weak texture, and ambiguous boundaries. More importantly, thick clouds may directly occlude land-cover content rather than only attenuating scene radiance. Once the underlying information is missing from the observation, an ASM-based dehazing model cannot faithfully recover it. To further support this limitation analysis, we report no-reference quality metrics for the cloud–haze coupled cases shown in Figure 11. Since these real cases do not have accurately aligned haze/cloud-free references, full-reference metrics such as PSNR and SSIM are not applicable. The no-reference results show that RDPC can improve perceptual quality to some extent, but the visual comparisons still reveal remaining artifacts around thick cloud-covered regions. These observations indicate that cloud–haze coupled restoration involves not only haze removal but also missing-content reconstruction. In future work, we will explore cloud-aware generative restoration with local structural similarity, where neighboring regions with similar textures or semantic patterns can guide the reconstruction of cloud-occluded land-cover structures.

## 5. Conclusions

In this paper, we proposed RDPC, a physics-driven and training-free framework for single RS image dehazing. RDPC revisits the atmospheric scattering model from the perspective of RS imaging, and builds the restoration process on three physical properties: RGB-space line convergence in local homogeneous regions, perpendicular guidance for transmission estimation, and ASM-consistent variation sparsity for joint refinement. Based on these properties, RDPC estimates atmospheric light without relying on explicit sky or dense haze regions, recovers transmission through local-to-global geometric modeling, and refines transmission and albedo guidance with variation-aware optimization.

Experiments on synthetic and real-world RS dehazing datasets show that RDPC achieves competitive and balanced performance in restoration fidelity, structure preservation, perceptual quality, and computational efficiency. Specifically, RDPC obtains PSNR/SSIM/LPIPS values of 19.11/0.7874/0.3854 on RSHaze, 20.57/0.8223/0.1007 on StateHaze1K, and 20.31/0.8072/0.1996 on LHID, respectively, with an average processing time of 0.101 s for a 512 × 512 image. For no-reference evaluation, RDPC also achieves BRISQUE/NIMA scores of 14.1484/5.1373, 5.1044/5.0615, and 13.2968/4.8318 on RSHaze, StateHaze1K, and LHID, respectively.

Nevertheless, RDPC still has limitations in cloud–haze coupled scenes, where thick clouds may occlude land-cover information that cannot be faithfully recovered by conventional ASM-based dehazing. In future work, we will explore cloud-aware generative restoration by distinguishing haze attenuation from cloud occlusion more explicitly. We also plan to introduce local structural similarity and semantic consistency constraints, so that neighboring regions with similar textures or land-cover patterns can guide the reconstruction of cloud-occluded structures. In addition, broader downstream evaluations, such as object detection, semantic segmentation, and land-cover classification, will be considered to more comprehensively assess the practical utility of RS image restoration methods. 

## Figures and Tables

**Table 1:** Ablation study for the two key terms in the joint optimization module, namely the albedo guidance (AG) term and the refined transmission (RT) term. PT denotes processing time.

Method	PSNR	SSIM	LPIPS	PT(s)
w/o AG	18.12	0.7725	0.2314	0.077
w/o RT	17.31	0.7576	0.2251	0.075
w/o AG&RT	15.57	0.6776	0.2799	0.064
RDPC	20.31	0.8072	0.1996	0.101

**Table 2:** Quantitative comparison between RDPC and other dehazing methods on RSHaze, StateHaze1K, and LHID using PSNR, SSIM, LPIPS, and processing time (PT). Best results are highlighted in bold, and second-best results are highlighted in red. The arrows indicate the preferred direction of each metric.

Method	RSHaze			StateHaze1K			LHID			PT(s)
	PSNR↑	SSIM↑	LPIPS↓	PSNR↑	SSIM↑	LPIPS↓	PSNR↑	SSIM↑	LPIPS↓	
ASTA	13.67	0.7200	0.3930	**22.72**	**0.8863**	0.1041	12.10	0.6416	0.3041	0.315
AU-Net	18.84	0.6993	0.4689	17.05	0.7208	0.2468	20.12	**0.8076**	0.2474	0.347
C2P	12.68	0.3870	0.5800	13.98	0.7155	0.2391	14.38	0.7225	0.2445	2.006
IDE	11.37	0.5080	0.5850	16.32	0.8150	0.1551	13.74	0.6638	0.2756	1.302
EMPF	15.68	0.7770	0.4280	13.53	0.7163	0.1892	18.32	0.7941	**0.1466**	0.583
IPC	13.06	0.5380	0.5490	18.94	0.8270	0.1465	15.13	0.6696	0.2884	4.841
LFD	13.97	0.7190	0.4760	10.10	0.6241	0.3912	17.04	0.7976	0.2062	0.117
RDPC	**19.11**	**0.7874**	**0.3854**	20.57	0.8223	**0.1007**	**20.31**	0.8072	0.1996	**0.101**

**Table 3:** Quantitative comparison between RDPC and other dehazing methods on RSHaze, StateHaze1K, and LHID using BRISQUE and NIMA. Lower BRISQUE and higher NIMA indicate better perceptual quality. Best results are highlighted in bold, and second-best results are highlighted in red. The arrows indicate the preferred direction of each metric.

Method	RSHaze		StateHaze1K		LHID	
	BRISQUE↓	NIMA↑	BRISQUE↓	NIMA↑	BRISQUE↓	NIMA↑
ASTA	37.3737	4.0137	6.9231	4.6474	27.6515	4.1786
AU-Net	20.2170	4.2427	5.5244	4.5171	14.7301	4.5053
C2P	21.2955	4.2501	5.9707	4.1716	25.3581	4.1019
IDE	14.6009	5.0727	8.3017	4.6723	31.7945	4.3702
EMPF	18.8086	4.8328	10.7292	4.3081	27.4470	4.2820
IPC	16.5124	4.7058	16.8720	4.9009	28.0718	4.8111
LFD	14.6944	4.8534	6.7615	3.9716	25.2492	4.2686
RDPC	**14.1484**	**5.1373**	**5.1044**	**5.0615**	**13.2968**	**4.8318**

**Table 4:** Quantitative comparison of detection confidence on two representative examples and the StateHaze1K dataset using YOLOv8. Case 1 and Case 2 correspond to the two detection examples shown in Figure 8. “–” indicates that the target is not detected. SBF denotes the soccer-ball field, while GTF denotes the ground-track field. For the StateHaze1K dataset, “Avg. SBF” and “Avg. GTF” denote the average detection confidence of soccer-ball fields and ground-track fields, respectively. Best results are highlighted in bold, and second-best results are highlighted in red.

Method	Case 1		Case 2	StateHaze1K	
	SBF	GTF	GTF	Avg. SBF	Avg. GTF
IDE	0.88	0.68	0.89	0.7317	0.7146
IPC	–	–	0.88	0.7074	0.6962
C2P	–	–	0.84	0.6849	0.6628
ASTA	0.81	–	**0.91**	0.7163	0.7015
AU-Net	0.84	0.76	0.88	0.7248	0.7093
LFD	–	–	–	0.6765	0.6639
EMPF	0.87	0.62	0.79	0.6812	0.6873
RDPC	**0.89**	**0.78**	0.88	**0.7469**	**0.7285**

**Table 5:** Detection results of different dehazing methods on two representative small-object cases and the DHID dataset using YOLOv8-OBB. Case 1 and Case 2 correspond to the two small-object detection examples shown in Figure 9. “Number” indicates the number of detected objects, and “Conf.” indicates the average detection confidence in each representative case. For the DHID dataset, “Total Number” denotes the total number of detected objects over all test images, whereas “Avg. Conf.” denotes the average confidence of all detected objects. Best results are highlighted in bold, and second-best results are highlighted in red.

Method	Case 1		Case 2		DHID	
	Number	Conf.	Number	Conf.	Total Number	Avg. Conf.
IDE	13	0.8001	7	0.7202	187	0.6736
IPC	13	0.7879	9	0.7099	305	0.7216
C2P	13	0.7895	9	0.6922	215	0.6742
ASTA	13	0.7739	7	0.7027	189	0.6738
AU-Net	13	0.7738	5	0.7111	164	0.6651
LFD	13	0.7907	5	0.6998	179	0.6829
EMPF	11	0.6921	8	0.6924	131	0.6647
RDPC	13	**0.8062**	9	**0.7281**	**320**	**0.7324**

## Data Availability

The data supporting the findings of this study are derived from publicly available remote sensing dehazing datasets, including RSHaze, StateHaze1K, LHID, RRSD300, and DHID, as cited in the manuscript. RSHaze, StateHaze1K, and LHID are used for quantitative evaluation with paired hazy/clear images, while RRSD300 provides real-world hazy remote sensing images for qualitative evaluation. DHID is used for downstream small-object detection evaluation. These datasets can be accessed through their original publications or official project repositories. No new public dataset was generated during this work. Additional experimental results or implementation details are available from the corresponding author upon reasonable request.

## Supplementary Materials

The following supporting information can be downloaded at: https://www.mdpi.com/article/10.3390/s26134026/s1, Figure S1: Visual comparison of the dehazing results with different values of θ.
