# Supplementary material for: Remote Sensing Image Dehazing via RGB-Space Physical Constraints
Source: Sensors (Basel). 2026 Jun 25;26(13):4026. doi: 10.3390/s26134026 (PMC13364281; doi:10.3390/s26134026)
Supplement: Supplementary file 1 [file sensors-26-04026-s001.zip › sensors-4335298-supplementary.pdf]

Supplementary Material

This supplementary material provides additional details and analyses to support the work. Specifically, we include the detailed derivation of Eq. (5), further explanation of the minimum information loss (MIL) guidance, and parameter analysis of θ used in the variation-degree estimation. These materials are intended to clarify the geometric motivation of the proposed method and to further justify the parameter setting adopted in the main experiments.

1. Detailed derivation of Eq. (5)

Under the ASM, the observed local darkest pixel I_i^d, the atmospheric light A, and the corresponding haze-free radiance are constrained on the same RGB-space line. Therefore, RDPC searches for the haze-removal guidance along the line determined by A and I_i^d, rather than modifying RGB values in an arbitrary RGB direction. Let this RGB-space line be parameterized as

$$l_i(s) = A + s(I_i^d - A),$$

where s is a scalar parameter. To obtain a lower-intensity guidance point while preserving the ASM-induced line constraint, we minimize the Euclidean distance from $l_i(s)$ to the origin:

$$s^* = \arg\min_s \|l_i(s)\|_2^2 = \arg\min_s \|A + s(I_i^d - A)\|_2^2 .$$

Taking the derivative with respect to s and setting it to zero gives

$$2(A + s^*(I_i^d - A))^T(I_i^d - A) = 0.$$

Thus,

$$s^* = -\frac{A^T(I_i^d - A)}{\|I_i^d - A\|_2^2}.$$

Substituting s^* into $l_i(s)$, we obtain

$$L_i^d = A - \frac{A^T(I_i^d - A)}{\|I_i^d - A\|_2^2}(I_i^d - A).$$

This is the perpendicular foot from the origin to the RGB-space line defined by A and I_i^d. It provides a minimum-intensity reference under the ASM-induced line constraint, thereby suppressing haze-induced brightness while preserving the physical color direction of the observed pixel. Therefore, the term “minimum-information-loss guidance” refers to reducing arbitrary RGB modification under the proposed geometric constraint.

2. *Parameter Analysis of θ.*

The parameter θ is used to balance the chromaticity-variance term and the texture-density term in the variation-degree estimation. To analyze its influence, we test different values of θ, including 0.5, 1.0, 1.5, 2.0, and 2.5, as shown in Fig. R1.

When θ is set to a relatively small value, such as 0.5 or 1.0, the restored images tend to be over-enhanced. Although haze is largely removed, some regions become unnaturally dark, and

local color distortion can be observed. This indicates that insufficient weighting of the texture-density term may lead to unstable block selection and overly aggressive restoration. In contrast, when θ is increased to 2.0 or 2.5, the dehazing effect becomes weaker, and visible haze remains in the restored images, especially in large homogeneous regions such as water surfaces and background areas.

Compared with these settings, θ = 1.5 achieves a better balance between haze removal and visual naturalness. It effectively reduces haze while preserving more reasonable brightness, color, and structural details. Therefore, θ is empirically fixed to 1.5 in all experiments.

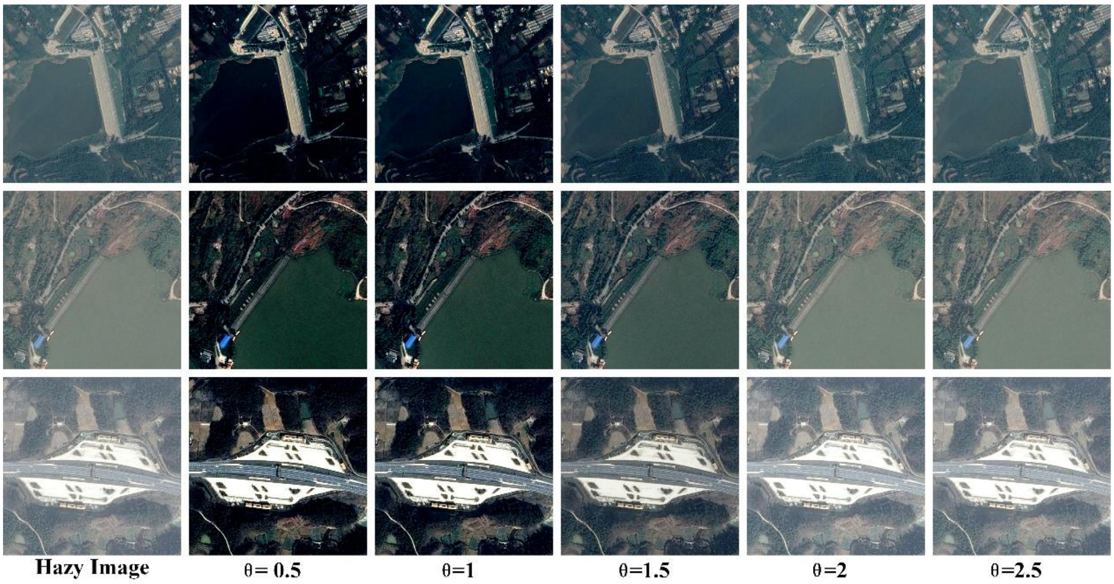

Figure S1: Visual comparison of the dehazing results with different values of θ.
